# YM155 decreases radiation-induced invasion and reverses epithelial–mesenchymal transition by targeting STAT3 in glioblastoma

**DOI:** 10.1186/s12967-018-1451-5

**Published:** 2018-03-23

**Authors:** Xin Zhang, Xuehai Wang, Ran Xu, Jianxiong Ji, Yangyang Xu, Mingzhi Han, Yuzhen Wei, Bin Huang, Anjing Chen, Qing Zhang, Wenjie Li, Jian Wang, Xingang Li, Chen Qiu

**Affiliations:** 1 Department of Neurosurgery, Qilu Hospital of Shandong University and Brain Science Research Institute, Shandong University, Jinan, 250012 People’s Republic of China; 2 Department of Otolaryngology, Weihai Municipal Hospital, Weihai, 264200 Shandong People’s Republic of China; 3 Department of Neurosurgery, Jining No. 1, People’s Hospital, Jining, 272011 China; 4 Department of Biomedicine, University of Bergen, 5009 Bergen, Norway; 5 Department of Radiation Oncology, Qilu Hospital of Shandong University, Jinan, 250012 People’s Republic of China

**Keywords:** Epithelial–mesenchymal transition, Glioblastoma, Radiation, STAT3, YM155

## Abstract

**Background:**

Radiotherapy constitutes a standard arm of therapy in the multimodal treatment of patients with glioblastoma (GBM). Ironically, studies have recently revealed that radiation can augment malignant progression, by promoting migration and invasion, which make the disease especially difficult to cure. Here, we investigated the anticancer effects of YM155, a purported radiosensitizer, in GBM cell lines.

**Methods:**

GBM cell lines U251 and U87 were treated with YM155 to assess cytotoxicity and activity of the molecule in vitro. Nude mice were implanted with cells to generate orthotopic xenografts for in vivo studies. Response of cells to treatment was examined using cell viability, immunofluorescence, wound healing, and the Transwell invasion assay. Molecules potentially mediating response were examined through western blot analysis, phospho-kinase arrays, and qPCR. Cells were transfected with siRNA knockdown and gene expression constructs to identify molecular mediators of response.

**Results:**

YM155 reduced viability of U251 and U87 cells and enhanced radiosensitivity through inhibition of homologous recombination. Besides, YM155 decreased invasion caused by radiation and led to expression changes in molecular markers associated with EMT. STAT3 was one of 10 molecules identified on a phosphokinase array exhibiting significant change in phosphorylation under YM155 treatment. Transfection with STAT3 siRNAs or expression constructs demonstrated that EMT changes were achieved by inhibiting the phosphorylation of STAT3 and were survivin-independent. Finally, combining YM155 and radiation in orthotopic xenografts reduced growth and prolonged overall survival of animals.

**Conclusions:**

YM155 decreased radiation-induced invasion in GBM cell lines in vitro and in vivo through inhibition of STAT3.

**Electronic supplementary material:**

The online version of this article (10.1186/s12967-018-1451-5) contains supplementary material, which is available to authorized users.

## Background

Glioblastoma (GBM) is the most aggressive primary tumor of the central nervous system and accounts for ~ 50% of all adult gliomas [1]. Current management is based on cytoreduction through a combination of surgery, radiation therapy, and chemotherapy. Despite this multidisciplinary approach to treatment, the prognosis of patients with GBM remains poor with a median overall survival of 9–15 months and a 2-year survival rate of 9–26% [2].

Radiotherapy, which kills cancer cells by causing DNA damage, is a highly cost-effective single-modality treatment [3]. However, studies have also reported that radiation can induce even more aggressive behavior in cancer cells [4–6], which further contributes to its failure in the treatment of GBM patients. Studies have found that epithelial–mesenchymal transition (EMT) is involved in the aggressive invasion of cancer cells after radiation [7–9]. EMT, a vital process in embryonic development, is believed to be utilized by cancer cells to increase mobility and invasiveness during metastasis [10, 11]. A hallmark of EMT is the downregulation of E-cadherin and the upregulation of the mesenchymal markers, such as N-cadherin and β-catenin. It has been well documented that cells that have undergone EMT withstand external insults better, leading to increase resistance to chemotherapy and radiotherapy [12, 13].

STAT3 is a cell proliferation-related transcription factor that regulates numerous apoptosis-related proteins including Bcl-2, Bcl-xL, Mcl-1, and cyclin D1 [14]. The activity of STAT3 has also been reported to correlate with the development of GBM [15]. STAT3 also displays an important role in EMT, and thus, downregulating the protein leads to reversal of EMT progress in cancer cells [16, 17]. Recently, STAT3 inhibitors have been shown to enhance the radiosensitivity of cancer cells [18, 19] and to reduce the malignant invasive ability induced by radiation [20, 21]. Thus, STAT3 inhibitors are promising candidates in combination with radiotherapy in the treatment of malignant cancers.

As a first-in-class small molecule inhibitor of survivin, YM155 selectively inhibited survivin expression at both mRNA and protein levels in the nanomolar range and exhibited anticancer activity in several types of neoplasms [22, 23]. Studies have also demonstrated that YM155 has a clear radiosensitizing effect in diverse cancers, including non-small cell lung cancer and esophageal squamous cell carcinoma [24, 25]. However, the mechanisms engaged by YM155 to radiosensitize GBM have not been fully investigated. In the present study, we examined the radiosensitizing effects of YM155 on GBM cells. We found that YM155 reversed EMT in glioma cells and prevented radiation-induced in vitro and in vivo invasion, and that YM155 might elicit these activities through inhibition of STAT3.

## Methods

### Cell culture

Human glioma cell lines U87 and U251 were purchased from the cell bank of the Chinese Academy of Sciences and were cultured in Dulbecco’s modified Eagle’s medium (ThermoFisher Scientific; Waltham, MA, USA) containing 10% fetal bovine serum (FBS; ThermoFisher Scientific), glutamine (4 mM), penicillin (10 U/mL), and streptomycin (100 mg/mL).

### Cell viability assay

Cell viability was assessed using Cell Counting Kit-8 (CCK-8, Dojindo; Kumamoto, Japan). U251 and U87 cells (1.0 × 10^4^ cells/well) were seeded into 96-well, flat-bottomed plates with DMEM containing 10% FBS and incubated at 37 °C overnight. YM155 (Selleck Chemicals; Houston, TX, USA) was dissolved in DMSO and diluted to working concentrations in culture medium. After 48 h, the cells were incubated for an additional 1 h at 37 °C with 10 μL of CCK-8 in 100 μL of serum-free DMEM. The absorbance at 450 nm was measured using a microplate reader (Bio-Rad; Hercules, CA, USA).

### EdU proliferation assay

U251 and U87 cells (2.5 × 10^4^ cells/well) were seeded into 24-well, flat-bottomed plates. After incubation at 37 °C overnight, cells were treated with increasing concentrations of YM155 for an additional 48 h in DMEM with 10% serum, and subsequently stained with EdU using the EdU incorporation assay kit (Ribobio; Guangzhou, China) according to the manufacturer’s instructions. EdU positive cells were counted from at least five random fields under fluorescence microscopy (Leica DMi8; Wetzlar, Germany).

### Colony formation assay

U251 and U87 cells (3 × 10^3^ cells/well) were plated in six-well plates. The adherent cells were treated with 5 nM YM155 or DMSO as control for 24 h before receiving one dose of 4 Gy at a dose rate of 1.8 Gy/min and subsequently incubated for 14 days. Colonies were rinsed with PBS, fixed in 4% paraformaldehyde, stained with 0.1% crystal violet and counted. Colony numbers were counted using the gel documentation system EAGLE EYETM II (UVP, LLC., Upland, CA, USA).

### Annexin V apoptosis assay

Apoptosis was evaluated using the FITC-annexin V/propidium iodide assay kit (BD Biosciences; San Jose, CA, USA). After treatment with YM155 or radiation, cells were collected by trypsin–EDTA, pelleted (1000 rpm for five flow cytometry in a minute), washed in ice-cold PBS, resuspended in the reagent containing annexin V-FITC and 1 µg/mL propidium iodide and Secondary antibodies were horseradish peroxidase-conjugated anti-mouse or anti-rabbit antisera incubated in the dark for 15 min. Cells were analyzed by flow cytometry using a C6 flow cytometer (BD Biosciences). A minimum of 20,000 cells per sample were analyzed.

### Western blot analysis

GBM cells were seeded in 6-well plates. After incubation overnight, cells were treated as indicated (YM155, radiation or combination treatment, or transfection) and then harvested at 48 h. Protein lysates (20 µg) were prepared in RIPA buffer, run on polyacrylamide gel electrophoresis, and transferred to PVDF membranes. Membranes were blocked with 5% skimmed milk in Tris-buffered saline containing 0.1% Tween-20, and subsequently incubated with primary and indicated secondary antibodies. Proteins on western blots where visualized using the Chemiluminescent Reagents Kit (Millipore, Billerica, MA, USA). Chemiluminescent signals were detected with the ChemiDoc XRS+ (Bio-Rad, Hercules, CA, USA) and quantified using Image Lab 3.0 software (Bio-Rad). Immunoblot analysis was performed according to the manufacturer’s instructions.

The following antibodies were used for Western blotting: phospho-histone H2A.X (Ser139), E-cadherin, N-cadherin, slug, β-catenin, Zeb1, STAT3, phospho-stat3 (Tyr705), Cyclin D1, and c-Myc (Cell Signaling Technology; Danvers, MA, USA); BRCA1 and phospho-stat3 (Ser727) (Abcam; Cambridge, MA, USA); GAPDH and Rad51 (Santa Cruz; Dallas, TX, USA). Secondary anti-mouse or anti-rabbit conjugated with horseradish peroxidase (Sigma-Aldrich; St. Louis, MO, USA).

### Homologous recombination (HR) assay

HR assays were performed with a kit (Norgen Biotek; Thorold, ON, Canada) according to the manufacturer’s instructions. Briefly, U251 or U87 cells were transfected with positive control plasmid or two HR dl plasmids (dl-1 and dl-2) on day 3 of DMSO or YM155 (5 nM) treatment. After 24 h, DNA was isolated using the Wizard genomic DNA purification kit (Promega; Madison, WI, USA). The recombined region was detected by qPCR performed with the supplied primers on a Roche LightCycler 480 II (Roche Applied Science; Indianapolis, IN, USA).

### Acti-stain 568 phalloidin staining

U251 and U87 cells cultured on coverslips were treated with DMSO or YM155 (5 nM) for 48 h. Cells were rinsed with PBS, fixed in 4% paraformaldehyde, and permeabilized with 0.3% Triton X-100. Cells were stained with acti-stain 568 phalloidin (Cytoskeleton; Denver, CO, USA) to label F-actin, and DAPI (Invitrogen; Carlsbad, CA, USA) was used to stain nuclei. Cells were examined under fluorescence microscopy (Leica DMi8).

### Cell migration and invasion assays

Cell migration was assessed in a wound healing assay. A silicone culture insert (Ibidi GmbH; Martinsried, Germany) was inserted into each well of a 4-well μ-slide (Ibidi GmbH), and U251 and U87 cells (70 μL at 4 × 10^5^ cells/mL) were added into each half of the culture inserts. After 24 h, the culture insert was removed, which resulted in a wound. Cells were rinsed with culture medium for three times, and DMSO or YM155 (5 nM) were added to the wells. Cell migration into the wound was examined at 0, 6, 12 and 24 h under bright field microscopy (Leica DMi8).

Invasion was measured using the Transwell Matrigel assay. Cells were seeded into 6-well plates. After treatment, cells were trypsinized, counted, and plated into a BD Biocoat Matrigel Invasion Chamber (pore size: 8 μm, 24-well; BD Biosciences) in serum free medium. The chemo-attractant was 10% FBS-containing medium (10%) added to the bottom wells. Invaded cells were fixed after 24 h, stained with crystal violet, and counted.

### Phospho-kinase antibody array

Phospho-kinases were analyzed using the Human Phospho-Kinase Array Kit (R&D Systems; Minneapolis, MN, USA) according the manufacturer’s instructions. Protein extracts were prepared from U251 cells treated with DMSO or YM155 (5 nM) for 48 h. Cell lysates were diluted and incubated overnight with the array membrane. The array was rinsed to remove unbound protein, incubated with an antibody cocktail, and developed using streptavidin–horseradish peroxidase and chemiluminescent detection reagents.

### Cell transfection

Cells were transfected twice with the same siRNA at a 24-h interval with lipofectamine 2000 (Invitrogen). The final concentration of siRNAs was 20 nM. Sequences for the siRNAs used were the following: survivin, 5′-GCATTCGTCCGGTTGCGCT-3′; STAT3, 5′-GUUCAUCUGUGUGACACCATT-3′; nontargeting siRNA controls, 5′-UUCUCCGAACGUGUCACGUTT-3′ (Genepharma, Shanghai, China). The STAT3 cDNA (KIAA1524) was purchased from the Addgene plasmid repository (http://www.addgene.org/). After transfection, U251 and U87 cells were cultured in the presence of G418 (0.8 mg/mL) for 8 weeks to select for stable transfectants. GBM cells that stably expressed STAT3 were used for treatment with YM155 and radiation.

### In vivo experiments

All animal protocols were approved by the ethics committee at the Shandong University (Jinan, China) and conducted according to the national regulations in China. Nude mice were anesthetized with 4% chloral hydrate (300 mg/kg) and placed in a stereotactic frame. Using aseptic surgical procedures, an incision was made in the parietal scalp, and a small burr hole was drilled 2.5 mm lateral to the bregma. U251 (1 × 10^6^ cells/mouse) were implanted 2.0 mm into the right striatum using a Hamilton syringe (Hamilton Co.; Reno, NV, USA). Two weeks later, mice were randomly divided into four groups (6 mice/group). Groups 1 and 2 were given two intratumoral injections of DMSO or YM155 (5 mg/kg) per week in a total of five injections. Group 3 was given three doses of localized irradiation (5 Gy) at days 15, 20, and 25 after implantation. Group 4 was irradiated three times following intratumoral injections of YM155 (5 mg/kg) per week in a total of 5 injections. Mice were sacrificed when central nervous system symptoms (such as poor ambulation, lethargy, and hunched posture) or weight loss of > 20% body mass developed. The mice were anesthetized with chloral hydrate and perfused transcardially with 4% paraformaldehyde in PBS. Whole brains were removed, post-fixed overnight in 4% paraformaldehyde in PBS, coronally sectioned into five slices, and paraffin embedded.

For subcutaneous GBM model, U87 cells were harvested by trypsinization, resuspended at 10^7^ cells/mL in a 1:1 solution of PBS/Matrigel (BD Biosciences, USA), and injected subcutaneously into the right shoulder of the nude mouse. Then mice (*n* = 24) were divided into four groups as described above. 4 days later, groups 1 and 2 were given intratumoral injections of DMSO or YM155 (5 mg/kg) every other day in a total of 5 injections. Group 3 was given three doses of localized irradiation (5 Gy) at days 5, 10, and 15 after implantation. Group 4 was irradiated three times following intratumoral injections of YM155 (5 mg/kg) per week in a total of 5 injections. Then tumor tissues were isolated 24 days after injection. Tumors were measured in three dimensions (a, b, c), and volume was calculated as abc × 0.52.

### Immunohistochemical analysis

Sections were cut (10 µm) from paraffin embedded tissues and stained with hematoxylin–eosin reagent or incubated with primary antibodies as indicated. The following primary antibodies were used for immunohistochemistry (IHC): phospho-stat3 (Tyr705) (Cell Signaling Technology), and phospho-stat3 (Ser727) (Abcam). Five representative images from each section were taken at 400× magnification using bright field microscopy (Leica DMi8). For each image, positive staining positive nuclei were counted using ImageJ software (National Institutes of Health; Bethesda, MD, USA). Positively staining cells are presented as a percentage of total number of cells counted.

### Statistical analysis

Unpaired t-tests were performed using SPSS software 13.0 (SPSS Inc., 2005; Chicago, IL, USA). Results are presented as the mean ± SE. *P* values < 0.05 were considered statistically significant.

## Results

### YM155 inhibits proliferation of GBM cells

**Fig. 1:**
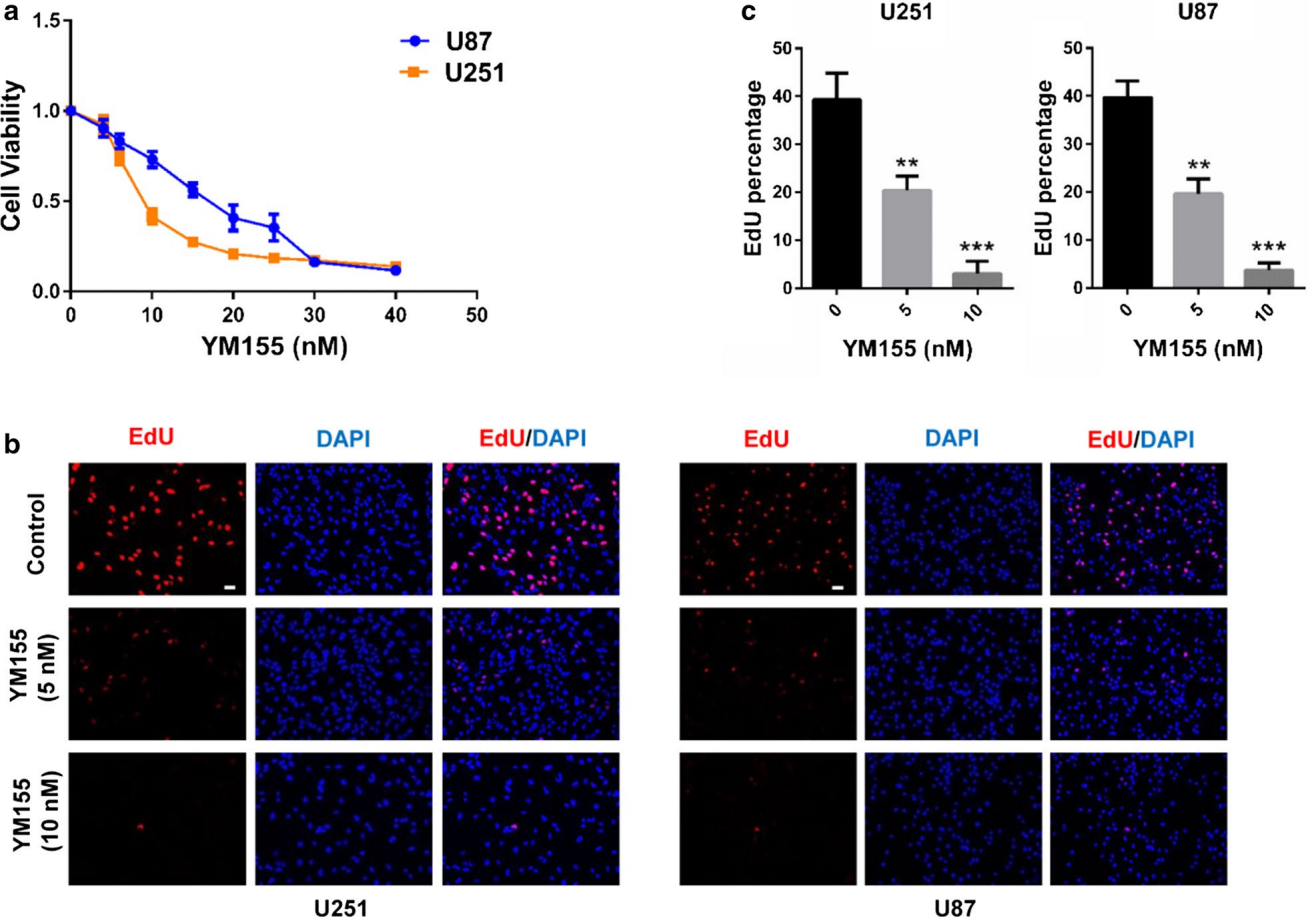
YM155 decreases cell viability and inhibits proliferation of GBM cells in vitro. **a** Cell viability of U251 and U87 cells after treatment for 48 h with increasing concentrations of YM155 using the CCK-8 assay. **b** Representative images of EdU incorporation performed on U251 and U87 cells treated with YM155 for 48 h at the concentrations indicated. **c** Graphic representation of the quantitation of EdU incorporation following treatment of cells with YM155 at the concentration indicated. **P < 0.01; ***P < 0.001; size bars = 50 µm

To begin to understand whether the drug elicited antitumor effects in glioma cells, U251 and U87 cell lines were treated with increasing concentrations of YM155, and cell viability and proliferation were assessed. Cell viability, as measured by the CCK-8 assay, of both U251 and U87 cells was decreased in the presence of YM155, and dose response curves yielded IC_50_ of 9 nM and 16 nM, respectively (Fig. 1a). Proliferation under YM155 treatment was examined using incorporation of EdU. EdU-positive cells decreased in a dose-dependent manner in response to YM155 (Fig. 1b, c). These data demonstrated that YM155 treatment led to decreased cell viability and proliferation.

### YM155 impairs homologous recombination

**Fig. 2:**
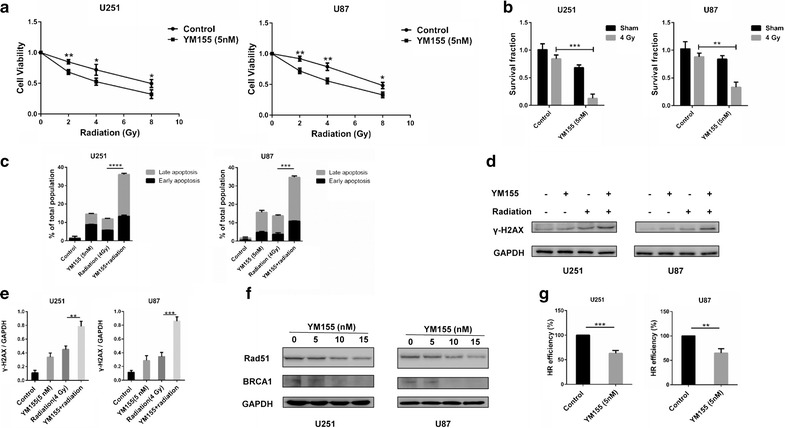
YM155 impairs HR. **a** Cell viability of U251 and U87 pretreated with DMSO or 5 nM YM155 after increased doses of radiation. **b** Bar graph representation of the number of colonies formed as related to control (DMSO). **c** Bar graph representation of the percentage of apoptosis as related to control (DMSO). Early apoptosis is measured using annexin V and late apoptosis is measured using PI. **d** Western blots to assess protein levels of γ-H2AX after treatment of indicated cells with DMSO, YM155, radiation and combined treatment (5 nM YM155 + radiation 4 Gy). **e** Quantitation of chemiluminescence of western blot analysis in (**d**). **f** Western blot analysis for protein levels of Rad51, BRCA1 and γ-H2AX in lysates prepared from cells treated with increasing concentrations of YM155. **g** HR efficiency of U251 and U87 cells after treatment with DMSO or 5 nM YM155 evaluated using qPCR to detect levels of a recombined fragment. *P < 0.05; **P < 0.01; ***P < 0.001; ****P < 0.0001

Previous studies found that YM155 was a highly effective radiosensitizer in the treatment of non-small cell lung cancer and esophageal squamous cell carcinoma [24, 25]. To determine whether YM155 enhanced radiation antitumor effects in glioma, viability of U251 and U87 cells treated with YM155 and then radiated was examined using the CCK-8 assay. Cell viability decreased significantly in cells treated with both radiation and YM155 (5 nM) compared to radiation alone (*P* < 0.05; Fig. 2a). We also found that combined treatment decreased colony formation and increased apoptosis significantly compared to radiation alone (Fig. 2b, c).

To begin to understand the underlying molecular mechanisms for this synergy, we first assessed the levels of phospho-histone H2A.X (Ser139), a biomarker for the presence of DNA damage, by western blot. The results of western blot analysis demonstrated that combining radiation with YM155 led to increased levels of phospho-histone H2A.X, over YM155 or radiation treatment alone (Fig. 2d, e). These results raised the possibility that DNA damage repair was attenuated in cells treated with YM155 in combination with radiation. To address this possibility, levels of proteins known to be involved in repairing DNA damage, Rad51 and BRCA1, were also examined by western blot. Cells treated with YM155 exhibited decreased levels of Rad51 and BRCA1 (Fig. 2f). Finally, we used a functional assay to test the efficiency of DNA repair in the presence of YM155. Recombinant plasmids were transfected into cells treated with YM155, and PCR was used to detect levels the novel recombined fragment. The results revealed that HR efficiency was reduced by 40% in cells treated with YM155 (Fig. 2g). Therefore, YM155 treatment could impair HR in GBM cells.

### YM155 inhibits increased invasion induced by radiation and reverses EMT in GBM cells

**Fig. 3:**
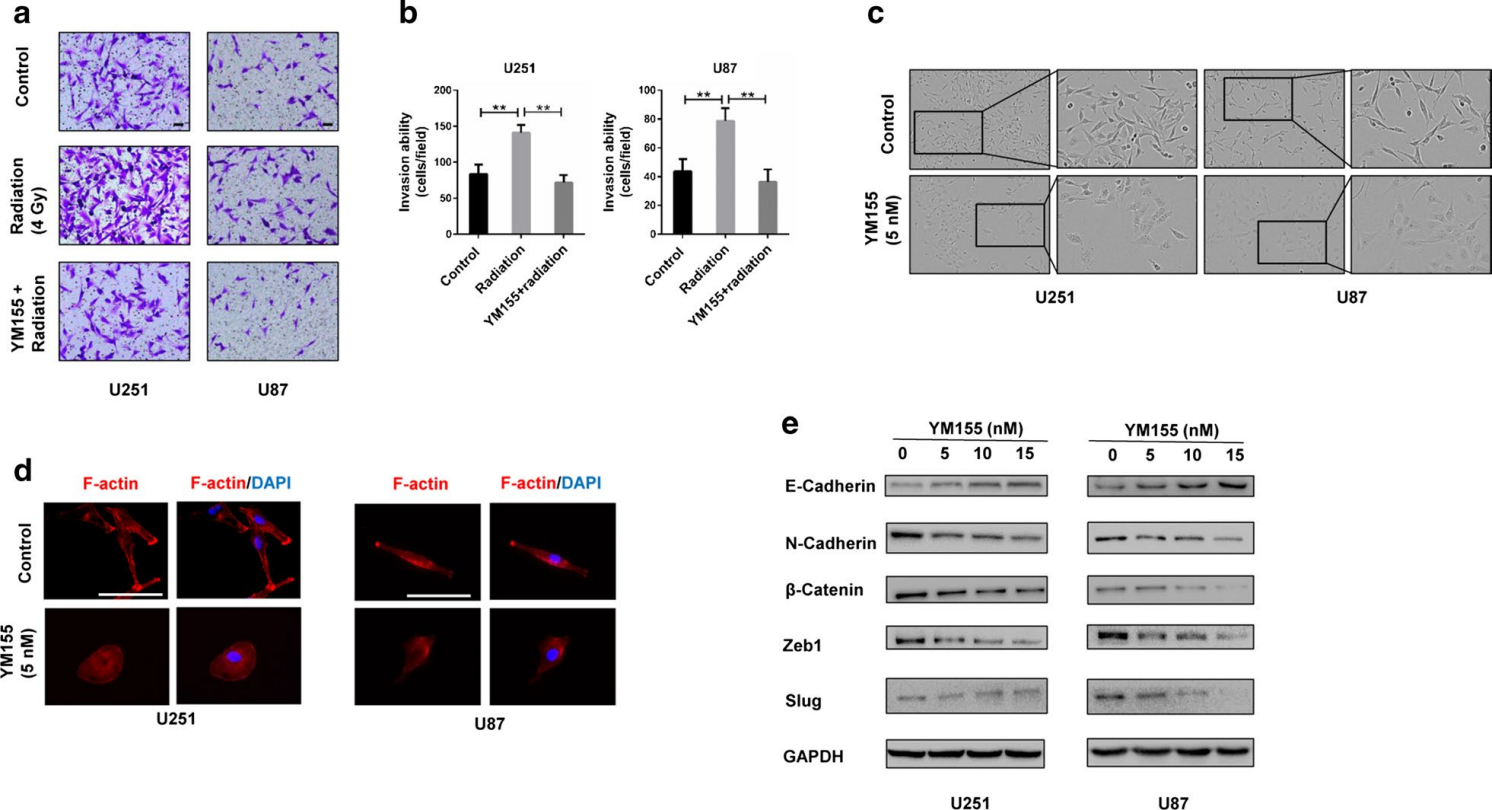
YM155 decreases invasion induced by radiation and reverses EMT in GBM cells. **a** Representative images of Transwell Matrigel assays from U251 and U87 cells in DMSO, radiation, and combination treatment (YM155 5 nM + radiation 4 Gy) fixed and stained with crystal violet. **b** Quantitation of migrated cell numbers from (**a**). **c** Morphology of U251 and U87 cells under bright field microscopy after treatment with vehicle or YM155. **d** Representative fluorescence images of phalloidin staining of DMSO and YM155 (5 nM) treated U251 and U87 cells. **e** Western blot analysis of E-cadherin, N-cadherin, β-catenin, Zeb1, and slug in U251 and U87 cells incubated with increasing concentrations of YM155. **P < 0.01; ***P < 0.001; size bars = 50 µm

Previous studies have demonstrated that radiation enhances invasion of tumor cells and YM155 has been reported to inhibit invasion of tumor cells [26]. We then try to explore the invasive ability of GBM cells after YM155 combined radiation treatment. After a single dose of 4 Gy, the number of invading cells increased by nearly twofolds over controls (Fig. 3a, b). However, combined treatment with YM155 reduced the number of invading cells to the level of controls (Fig. 3a, b). These data showed that YM155 could inhibit increased invasion induced by radiation.

To explore the underlying mechanisms, we found that after treatment with YM155, the morphology of U251 and U87 cells changed from a shuttle shape to a flat rounded shape (Fig. 3c). We then stained cells with phalloidin, a high-affinity F-actin probe, to examine actin cytoskeletal organization before and after treatment with YM155. Using fluorescence microscopy, differences in the morphology and actin cytoskeletal organization showed that YM155 led to actin filament reorganization (Fig. 3d). These data suggested that YM155 might cause EMT reversal [27].

Reversal of EMT under YM155 treatment was also evident through examination of proteins levels of several markers associated with EMT. By western blot, we found that YM155 led to increased levels of E-cadherin, an important epithelial marker, but decreased N-cadherin and β-catenin, two important mesenchymal markers (Fig. 3e). Slug and ZEB1, two transcriptional repressors of E-cadherin transcription, were also decreased after YM155 treatment (Fig. 3e).

To test cell motility in the presence of YM155, we used a wound healing assay. The wound healing rate was decreased by 50% under YM155 treatment in both U251 and U87 cells (Additional file 1: Figure S1). Taken together, these data indicated that YM155 inhibits increased invasion induced by radiation and reversed some features of EMT in GBM cells.

### STAT3 mediates EMT reversal induced by YM155

**Fig. 4:**
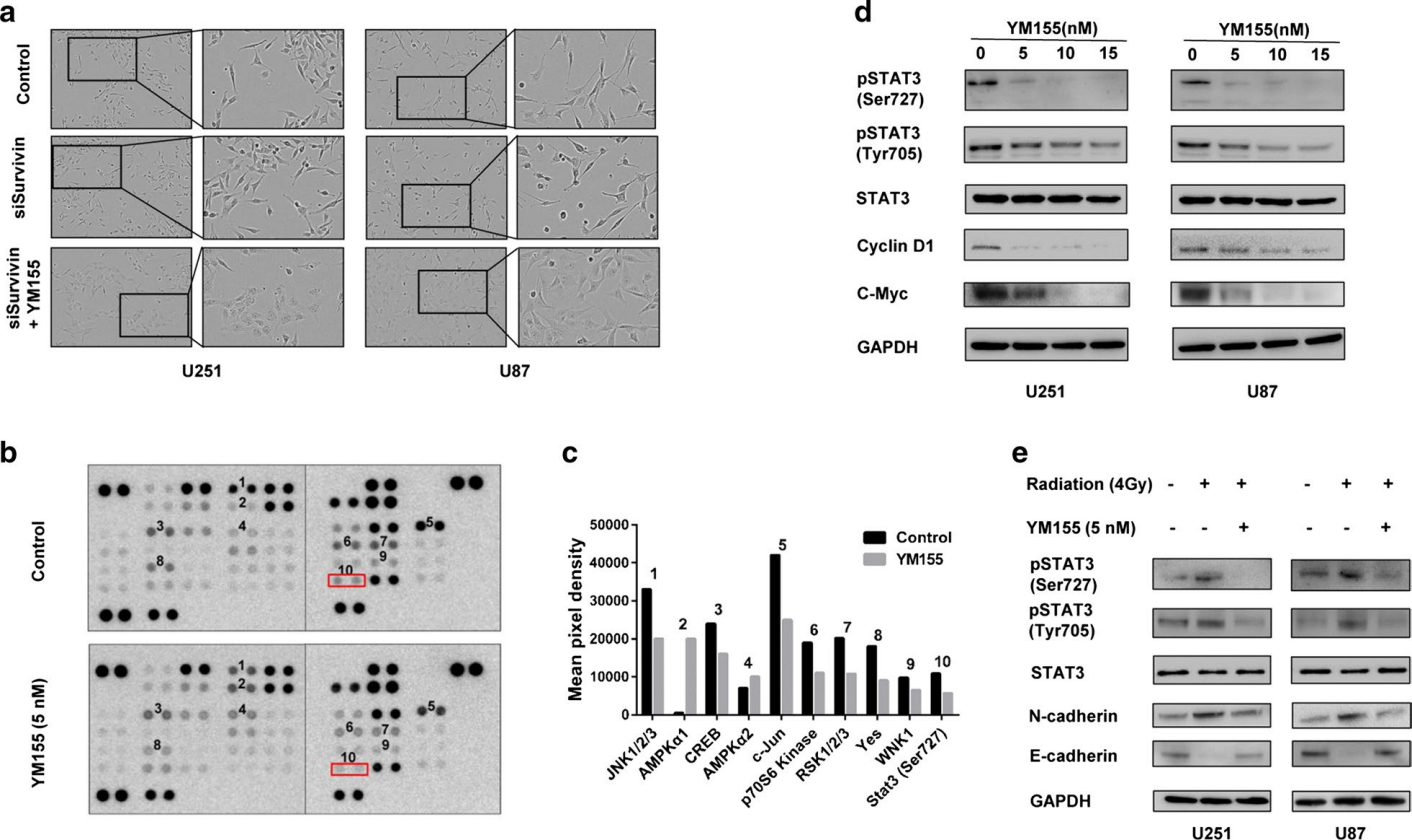
STAT3 mediates EMT reversal induced by YM155 independently of survivin. **a** Morphology of U251 and U87 cells before and after knockdown with survivin siRNA, or knockdown with YM155 treatment. **b** Phospho-kinase array of 43 phosphorylated kinases in U251 cells treated with 5 nM YM155 relative to DMSO control. **c** Graphic representation of the quantitation of 10 molecules with the most significant difference in phosphorylation status between YM155 treatment and control. **d** Western blot analysis of pSTAT3 (Ser727), pSTAT3 (Tyr705), STAT3, Cyclin D1 and c-Myc of U251 and U87 cells incubated with increasing concentrations of YM155. **e** Western blot analysis of pSTAT3 (Ser727), pSTAT3 (Tyr705), STAT3, N-cadherin and E-cadherin in U251 and U87 cells treated with DMSO, radiation, and combination treatment (YM155 5 nM + radiation 4 Gy)

YM155 is a molecule known to target survivin. We therefore examined the possibility that YM155 might reverse EMT through inhibition of survivin. Cells were transfected with survivin siRNAs and examined for changes in morphology. No change in the morphology of U251 or U87 cells was apparent with knockdown. However, YM155 treatment of cells transfected with survivin siRNAs (knocking down efficiency was verified by Western blot analysis in Additional file 2: Figure S2) still caused a morphological change similar to YM155 treatment alone (Fig. 4a).

These results indicated that EMT changes induced by YM155 might be mediated by kinases other than survivin. To identify candidate kinases mediating YM155 effects on EMT, we assessed the levels of 43 phosphorylated kinases in U251 cells exposed to 5 nM YM155 relative to control (DMSO) using a phospho-kinase array. Several kinases (*n* = 10) changed significantly under YM155 treatment (Fig. 4b, c). One of the kinases, STAT3, plays an important role in EMT. We further validated this result by western blot. Phospho-STAT3 (Ser727) levels were decreased significantly at very low dosage of YM155 (Fig. 4d). Phospho-STAT3 (Tyr705) was also decreased in a dose-dependent manner with YM155, although no change had been detected on the array (Fig. 4d). Proteins downstream of STAT3, cyclin D1 and c-Myc, were also decreased in a dose-dependent manner in response to YM155 treatment (Fig. 4d). Finally, although radiation treatment led to increases in phospho-STAT3 (Ser727) and phospho-STAT3 (Tyr705), combination treatment led to decreased levels compared to controls (Fig. 4e). We also found that YM155 reversed the up-regulation of N-cadherin and down-regulation of E-cadherin induced by radiation (Fig. 4e). Therefore, YM155 appeared to reverse radiation-induced EMT through inactivation of STAT3.

**Fig. 5:**
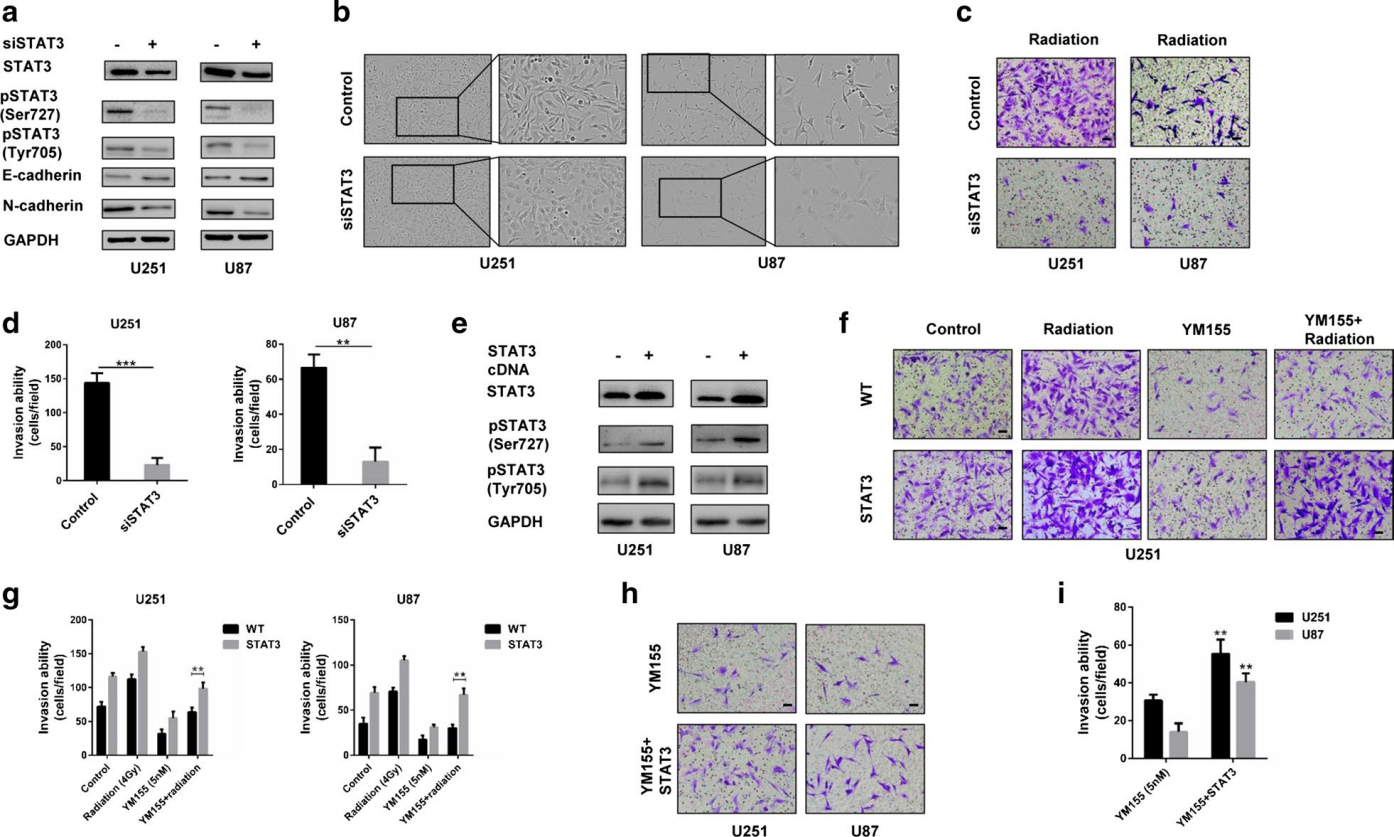
STAT3 mediates EMT reversal induced by YM155. **a** Western blot analysis of pSTAT3 (Ser727), pSTAT3 (Tyr705), STAT3, E-cadherin, and N-cadherin in U251 and U87 cells before and after knockdown with STAT3 siRNA. **b** Morphology of U251 and U87 cells before and after STAT3 siRNA knockdown. **c** Crystal violet staining of Transwell Matrigel assay for U251 and U87 cells control or transfected with STAT3 siRNA after radiation treatment. **d** Quantitation of migrating cell number in experiments from (**c**). **e** Western blot analysis of pSTAT3 (Ser727), pSTAT3 (Tyr705) and STAT3 in lysates prepared from control cells and cells stably expressing STAT3. **f** Crystal violet staining of Transwell Matrigel assay from control or stable ectopic expression of STAT3 U251 cells after control, radiation (4 Gy), YM155 (5 nM) and combination treatment (5 nM YM155 + 4 Gy radiation). **g** Quantitation of migrating cell number in experiments from (**f**). **h** Crystal violet staining of Transwell Matrigel assay from YM155 treated U251 and U87 cells or stable ectopic expression of STAT3 U251 and U87 cells following YM155 treatment. **i** Quantitation of migrating cell number in experiments from (**h**). **P < 0.01; ***P < 0.001; size bars = 50 µm

To further validate these findings, we transfected cells with STAT3 siRNAs and examined levels of EMT associated proteins by western blot. Knockdown of STAT3 led to increased E-cadherin but decreased N-cadherin levels (Fig. 5a). Furthermore, the morphology of U251 and U87 changed from a shuttle shape to a flat shape (Fig. 5b). Knockdown of STAT3 also decreased the invasive ability of GBM cells treated with radiation (Fig. 5c, d). When STAT3 was stably overexpressed in U251 or U87, the number of cells invading Matrigel-coated membranes increased significantly compared to parental U251 and U87 cells, even after combined treatment (Fig. 5e–g, and Additional file 3: Figure S3). Finally, we also found that expressing STAT3 in YM155 treated cells increased cell invasion again (Fig. 5h, i). These results indicated that YM155 reversed some features of EMT in GBM cells through inactivation of STAT3.

### YM155 enhances the antitumor effect of radiotherapy in vivo

**Fig. 6:**
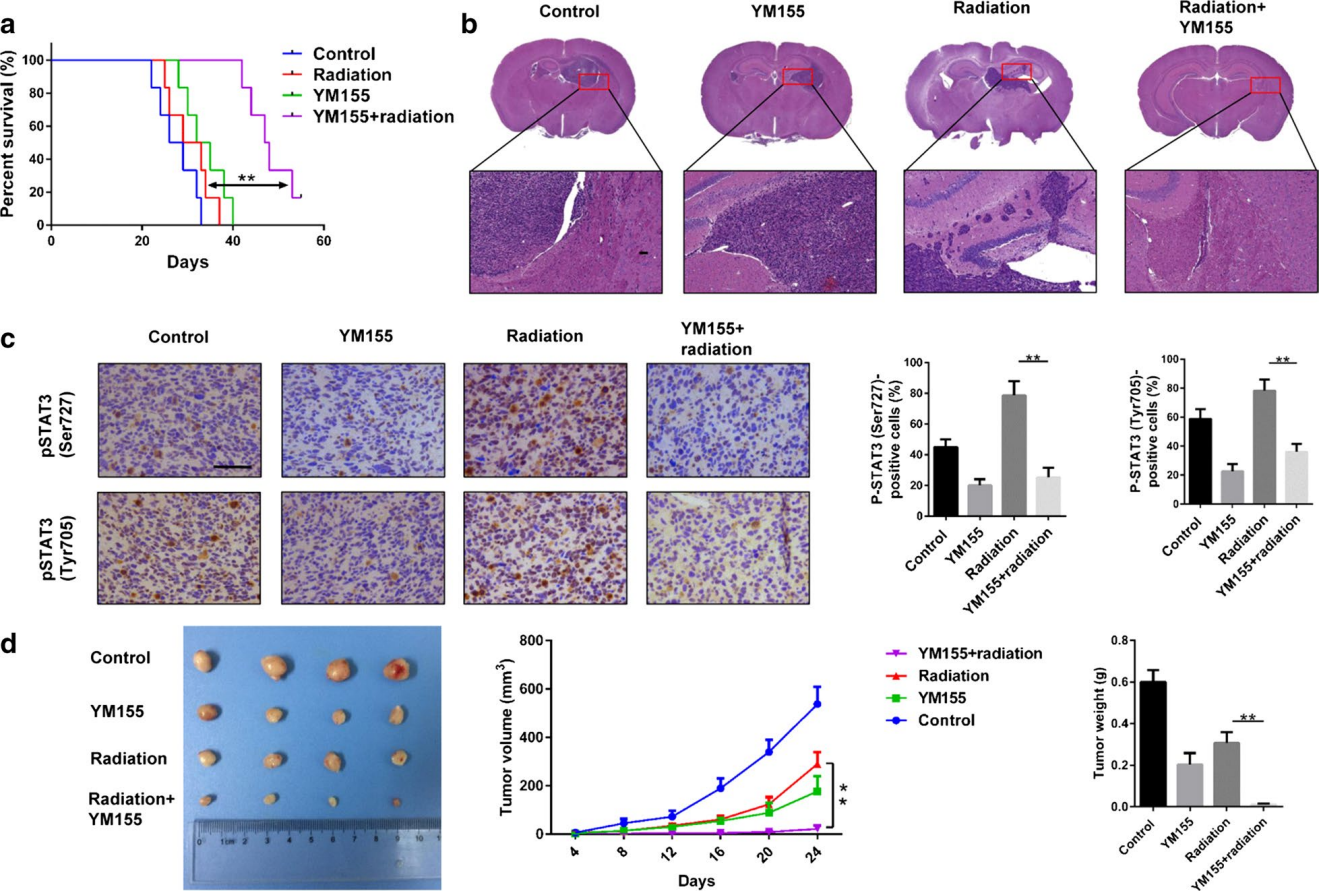
YM155 increases the antitumor effect of radiotherapy in vivo. **a** Survival curve of animals implanted with U251 cells in the four different treatment groups indicated. **b** Representative images of hematoxylin and eosin staining of brain sections from animals in each of the four different treatment groups indicated. **c** IHC for pSTAT3 (Ser727) and pSTAT3 (Tyr705) in xenograft sections from animals in each of the four different treatment groups. Quantitation of positive staining nuclei. Positively staining cells are presented as a percentage of total number of cells counted. **P < 0.01; size bars = 50 µm. **d** Representative images of subcutaneous tumors with U87 cells. Tumor volume and tumor weight were measured. **P < 0.01

To test the antitumor effect of the combination treatment in vivo, we established an orthotopic glioma model with U251 cells. To ensure drug delivery, we chose to administer YM155 intratumorally. Nude mice treated with combination therapy exhibited significantly prolonged overall survival compared to all other treatment groups (YM155 + radiation vs. control, *P* < 0.001; YM155 + radiation vs. YM155, *P* < 0.001; YM155 + radiation vs. radiation, *P* < 0.01; Fig. 6a). Tumor borders in radiation treatment group appeared more invasive, exhibiting many satellite foci. In contrast, under combined treatment, tumors were more circumscribed (Fig. 6b).

IHC performed on sections from xenografts demonstrated that phospho-STAT3 protein levels in vivo were consistent with the results performed on cells in vitro. Xenografts that had been irradiated exhibited increased expression of phospho-STAT3 (Ser727) and phospho-STAT3 (Tyr705), which were decreased significantly under combined treatment (Fig. 6c). These data indicated that the synergistic antitumor effect of the combined therapy was mediated in part through inhibition of STAT3.

With another animal model in which U87 cells were implanted subcutaneously, we found that the combined therapy decreased tumor volume and tumor weight significantly compared with other groups (Fig. 6d).

## Discussion

Radiotherapy is a mainstay treatment modality after surgical resection in GBM patients. It mainly kills cancer cells by causing DNA damage. However, many factors, such as DNA damage repair, cell cycle arrest and autophagy, lead to radioresistance in tumors [28]. Even worse is the discovery that radiation might actually enhance malignant progression in cancer cells, further contributing to the failure of radiotherapy in the clinic [4–6]. Accumulating evidence supports YM155 as a radiosensitizer of high efficiency [24, 25]. Besides, previous study showed that YM155 inhibited invasion in glioma cells [26]. Here, we not only found that YM155 increased the radiosensitivity of GBM cells by impairing HR, but we also observed YM155 inhibited invasion induced by radiation. The changes in the morphology and actin cytoskeletal organization of U251 and U87 were reminiscent of the process of EMT. These changes were also evident in the levels of several EMT markers indicating that YM155 reversed EMT in GBM cells. Such results render YM155 a molecule of interest in the treatment of human glioma, especially in the context of radiotherapy.

However, YM155 activity was mediated not only through survivin, the putative target of the drug; inhibition of survivin through siRNA knockdown, for example, did not induce EMT changes in the morphology of GBM cells. Using a phospho-kinase antibody array kit, we also identified STAT3 (decreased phosphorylation at Ser727) as a potential mediator of YM155 activity. STAT3 is a transcription factor, contributing to diverse biological processes, including tumor cell proliferation, migration, invasion and survival. Thus, STAT3 is an ideal target for cancer therapy [29, 30]. Our results are consistent with previous observations that STAT3 also plays an important role in promoting EMT [8, 16] and thus invasion and migration of tumor cells. We found that knockdown with STAT3 siRNA induced an epithelial-like morphology in U251 and U87 cells, which was consistent with changes in EMT markers at the molecular level. STAT3 knockdown also attenuated increased invasion of glioma cells induced by radiation. Finally, stable overexpression of STAT3 enabled invasion of GBM cells, despite treatment with YM155.

We identified YM155 as a promising radiosensitizer in GBM based on its ability to impair HR and reverse EMT. While YM155 does not easily penetrate the blood brain barrier [31], which is not like the antipsychotic we have reported before [32], we chose to administer YM155 intratumorally and found that the drug also inhibited invasion in vivo. More studies are needed to demonstrate efficacy in vivo combined with radiation. Nanotechnology may serve as the platform to enable further investigation of YM155 in animal models and ultimately in the clinic [33].

## Conclusions

The main finding of this study is that YM155 decreased radiation-induced invasion and reversed epithelial–mesenchymal transition by targeting STAT3 in glioblastoma. Our study revealed a potential mechanistic basis and provided theoretical support for radiosensitivity induced by YM155 in human glioma cells. Whether YM155 elicits anticancer effects through similar mechanisms in other neoplasms will be of interest for future investigations.

## Additional files

**Additional file 1: Figure S1.** YM155 decreases migration in GBM cells. (a) Representative images of wound healing assays for U251 and U87 cells incubated with DMSO or 5 nM YM155. (b) Graphic representation for quantitation of wound healing assays in (A). ***P < 0.001.

**Additional file 2: Figure S2.** Western blot analysis of survivin after knocking down survivin with siRNA.

**Additional file 3: Figure S3.** Crystal violet staining of Transwell Matrigel assay from control or stable ectopic expression of STAT3 U87 cells after control, radiation (4 Gy), YM155 (5 nM) and combination treatment (5 nM YM155 + 4 Gy radiation).

## Abbreviations

CCK-8: cell counting kit-8
DMSO: dimethyl sulfoxide
EMT: epithelial–mesenchymal transition
FBS: fetal bovine serum
GBM: glioblastoma
HR: homologous recombination
IHC: immunohistochemistry
PVDF: polyvinylidene fluoride
RIPA: radioimmunoprecipitation
