# Correction to: YM155 decreases radiation-induced invasion and reverses epithelial–mesenchymal transition by targeting STAT3 in glioblastoma

**DOI:** 10.1186/s12967-021-03017-8

**Published:** 2021-09-27

**Authors:** Xin Zhang, Xuehai Wang, Ran Xu, Jianxiong Ji, Yangyang Xu, Mingzhi Han, Yuzhen Wei, Bin Huang, Anjing Chen, Qing Zhang, Wenjie Li, Jian Wang, Xingang Li, Chen Qiu

**Affiliations:** 1 Department of Neurosurgery, Qilu Hospital of Shandong University and Brain Science Research Institute, Shandong University, Jinan, 250012 People’s Republic of China; 2 Department of Otolaryngology, Weihai Municipal Hospital, Weihai, 264200 Shandong People’s Republic of China; 3 Department of Neurosurgery, Jining No. 1, People’s Hospital, Jining, 272011 China; 4 Department of Biomedicine, University of Bergen, 5009 Bergen, Norway; 5 Department of Radiation Oncology, Qilu Hospital of Shandong University, Jinan, 250012 People’s Republic of China

## Correction to: J Transl Med (2018) 16:79 10.1186/s12967-018-1451-5

After publication of the original article [1], it was noticed that the images of WT (YM155+Radiation) in Fig. 5f and U251 (YM155+STAT3) in Fig. 5h were mistakenly duplicated. This error also affected supplementary figure 3. The original and updated figures are published in this correction article.

The updated figures (Figs. 1, 2, 3, 4) do not change the validity of the scientific conclusion drawn in this paper.

**Fig. 1:**
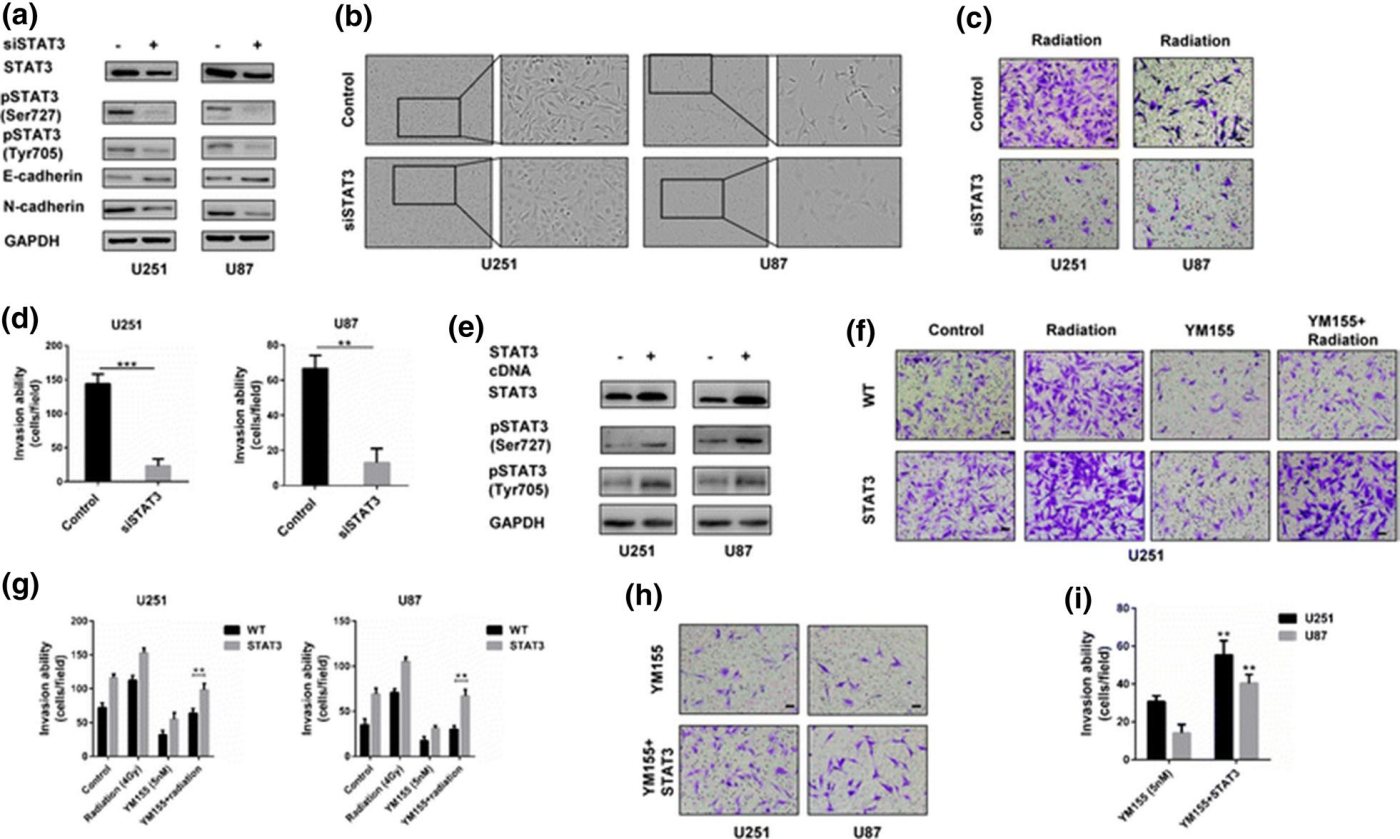
Original publication of figure 5h

**Fig. 2:**
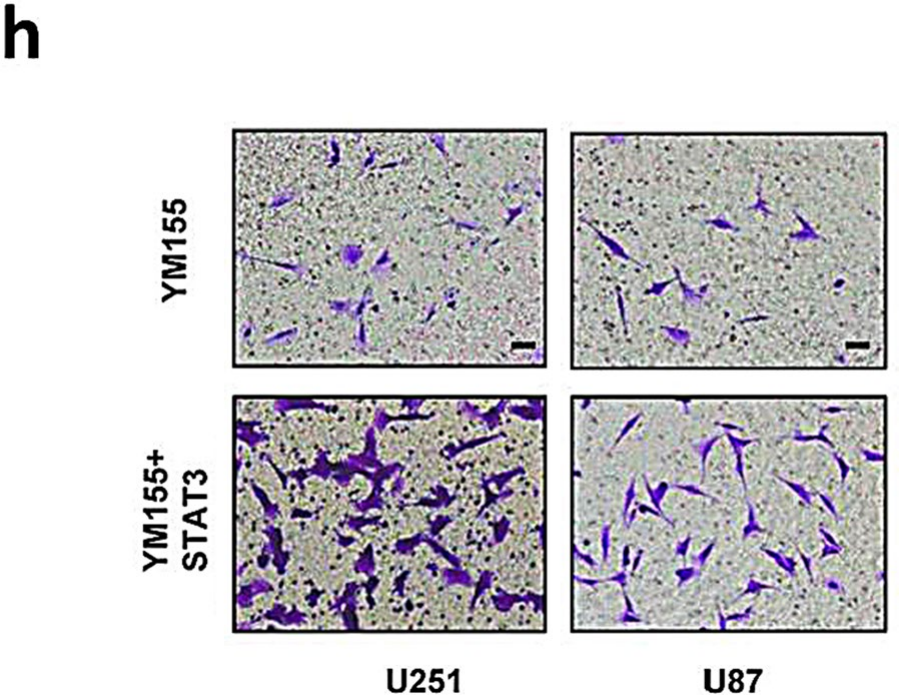
Updated figure 5h

**Fig. 3:**
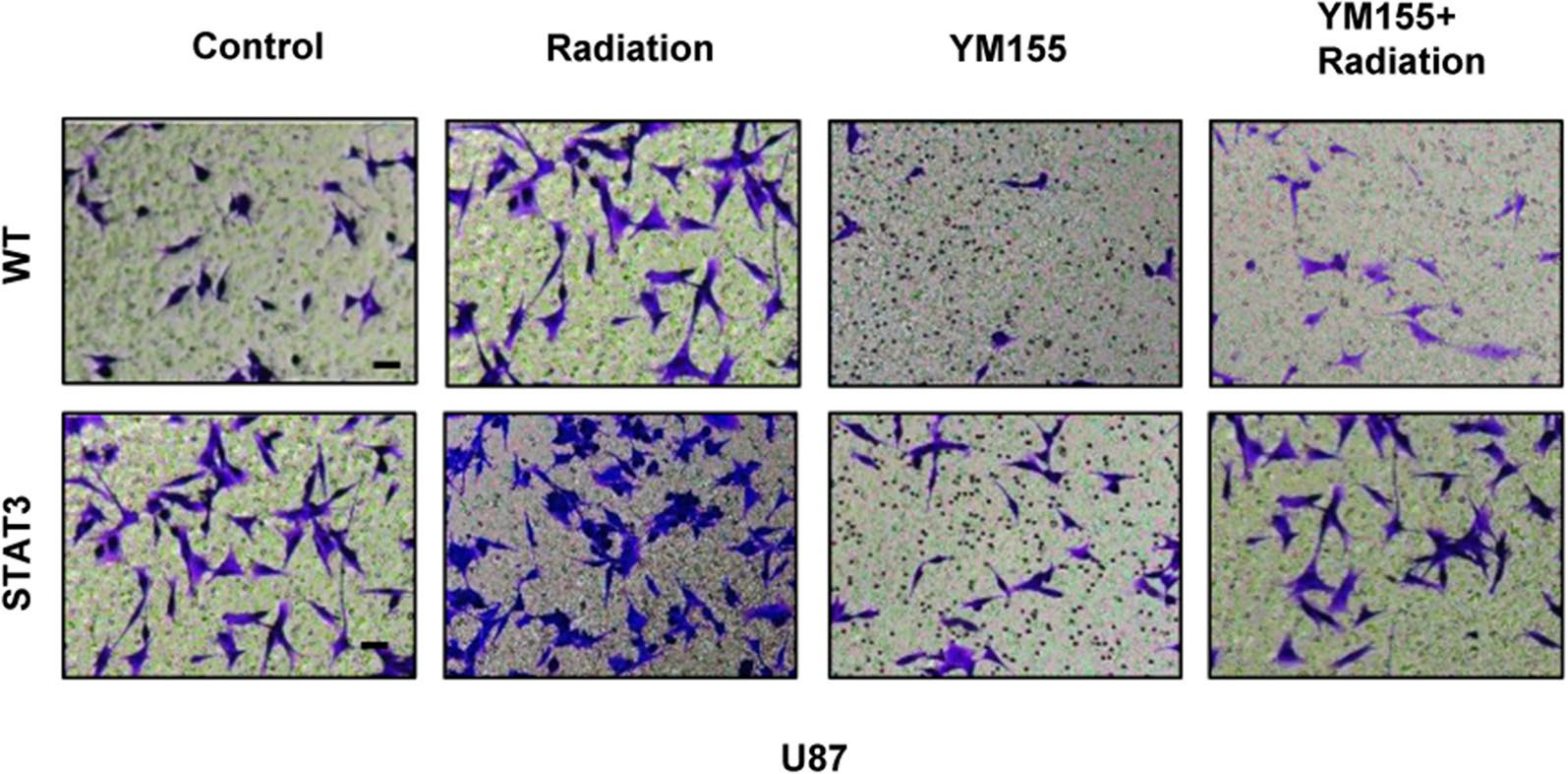
Original publication of supplementary figure 3

**Fig. 4:**
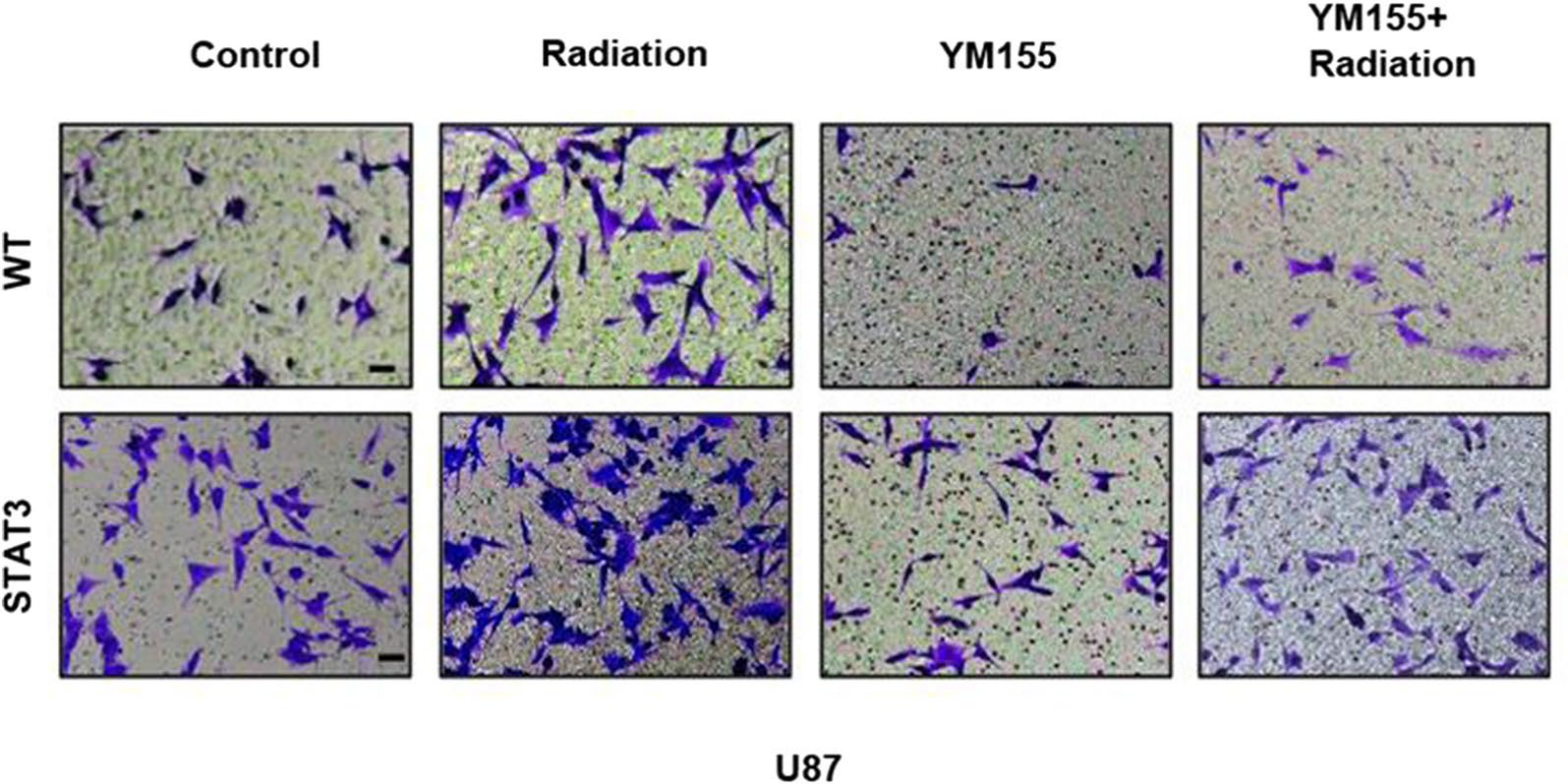
Updated supplementary figure 3
