# Supplementary figures and images for: YM155 decreases radiation-induced invasion and reverses epithelial–mesenchymal transition by targeting STAT3 in glioblastoma
Source: J Transl Med. 2018 Mar 23;16:79. doi: 10.1186/s12967-018-1451-5 (PMC5865331; doi:10.1186/s12967-018-1451-5)

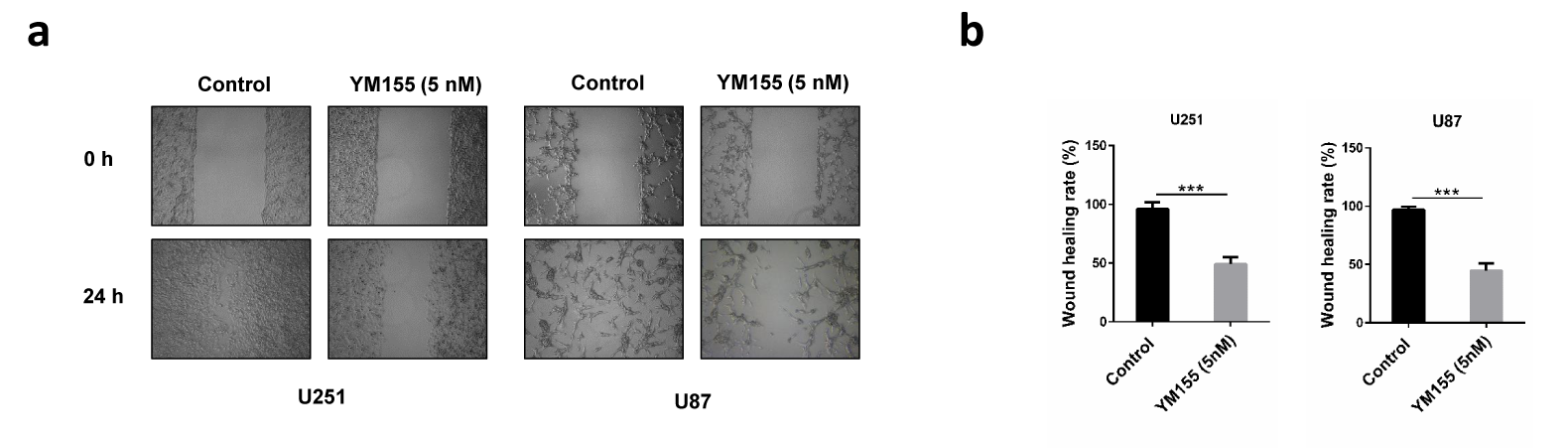

Supplement: Supplementary file 1 — Additional file 1: Figure S1. YM155 decreases migration in GBM cells. (a) Representative images of wound healing assays for U251 and U87 cells incubated with DMSO or 5 nM YM155. (b) Graphic representation for quantitation of wound healing assays in (A). ***P < 0.001. [file 12967_2018_1451_MOESM1_ESM.tif]

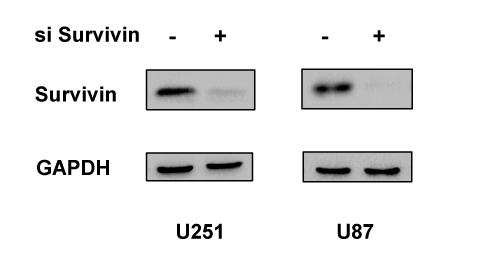

Supplement: Supplementary file 2 — Additional file 2: Figure S2. Western blot analysis of survivin after knocking down survivin with siRNA. [file 12967_2018_1451_MOESM2_ESM.tif]

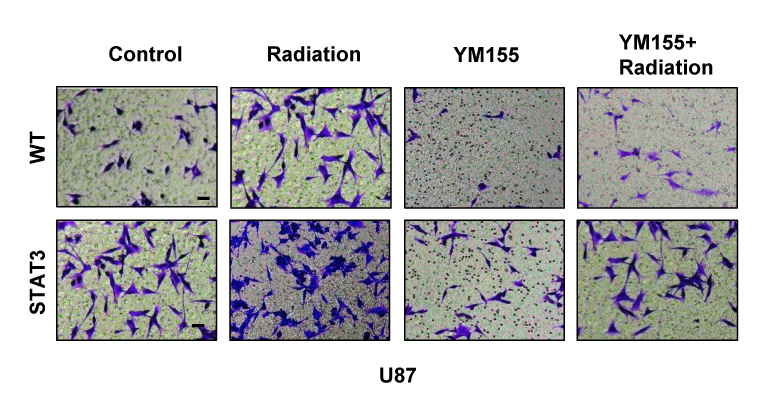

Supplement: Supplementary file 3 — Additional file 3: Figure S3. Crystal violet staining of Transwell Matrigel assay from control or stable ectopic expression of STAT3 U87 cells after control, radiation (4 Gy), YM155 (5 nM) and combination treatment (5 nM YM155 + 4 Gy radiation). [file 12967_2018_1451_MOESM3_ESM.tif]
